# Supplementary material for: Deep Eutectic Solvent-Based Ultrasound-Assisted Strategy for Simultaneous Extraction of Five Macamides from Lepidium meyenii Walp and In Vitro Bioactivities
Source: Foods. 2023 Jan 5;12(2):248. doi: 10.3390/foods12020248 (PMC9858098; doi:10.3390/foods12020248)
Supplement: Supplementary file 1 [file foods-12-00248-s001.zip › foods-2050016-supplementary.pdf]

Article

Deep Eutectic Solvent-Based Ultrasound-Assisted Strategy for Simultaneous Extraction of Five Macamides from *Lepidium meyenii* Walp and In Vitro Bioactivities

Keke Li [1], Zhongyu Li [1], Lei Men [1], Jiwen Li [1,2] and Xiaojie Gong [1,2,*]

[1] College of Life Sciences, Key Laboratory of Biotechnology and Bioresources Utilization, Dalian Minzu University, Ministry of Education, Dalian 116600, China
[2] School of Biological Engineering, Dalian Polytechnic University, Dalian 116034, China
* Correspondence: gxjclr@163.com; Tel.: +86-411-87656057

Supplementary Information

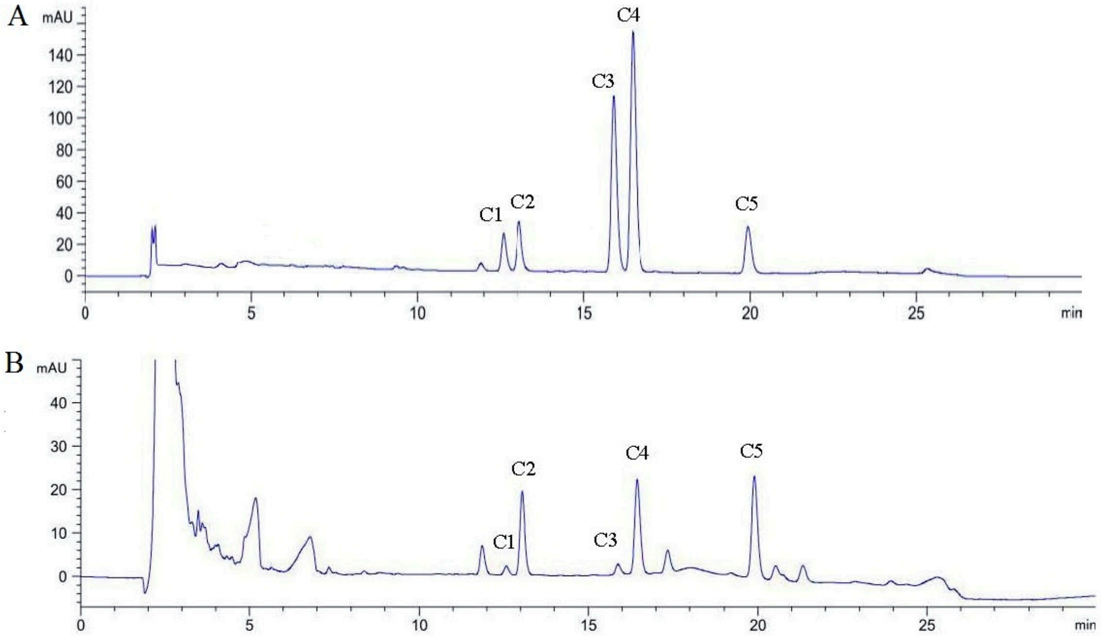

Figure S1. Representative HPLC-UV chromatographic profiles of standards mixture (A) and extract by DES-7 with 20% water content (B).

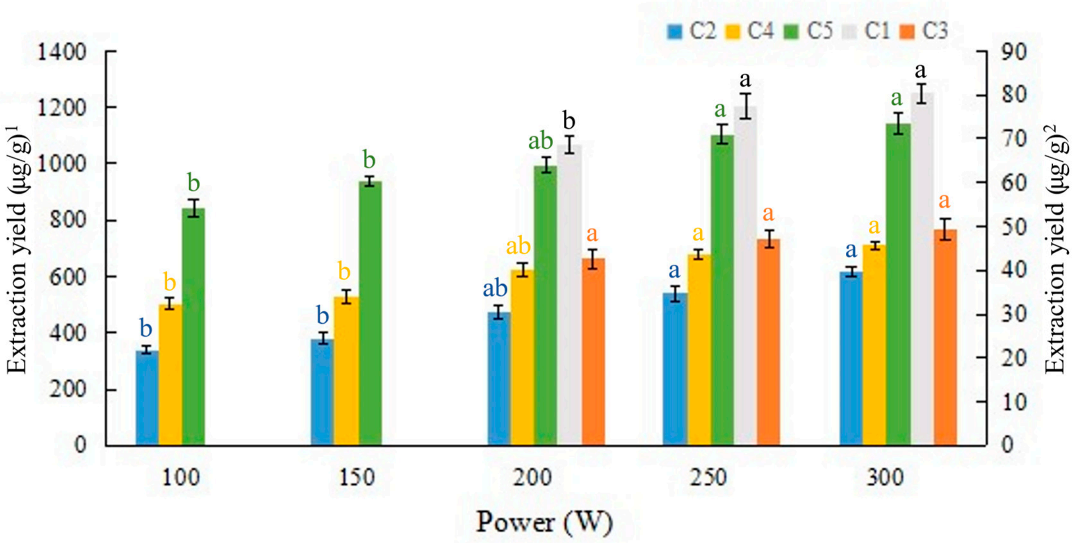

Figure S2. Effect of ultrasound power on the yields of five target macamides. Different lowercase in the same color of column represent the significant difference at $p < 0.05$. ([1] Extraction yields of C2, C4 and C5; [2] Extraction yields of C1 and C3).

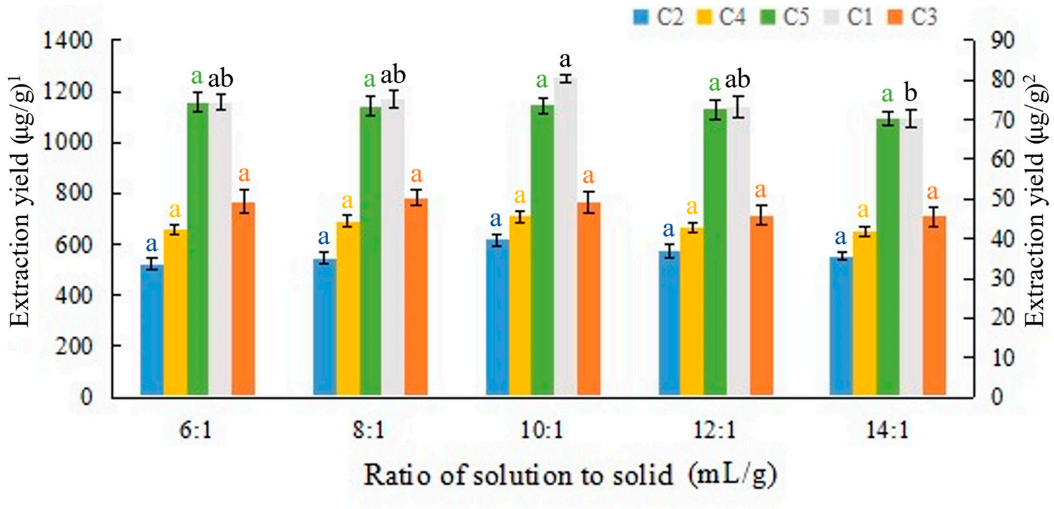

Figure S3. Effect of ratio of solution to solid on the yields of five target macamides. Different lowercase in the same color of column represent the significant difference at $p < 0.05$. ([1] Extraction yields of C2, C4 and C5; [2] Extraction yields of C1 and C3).

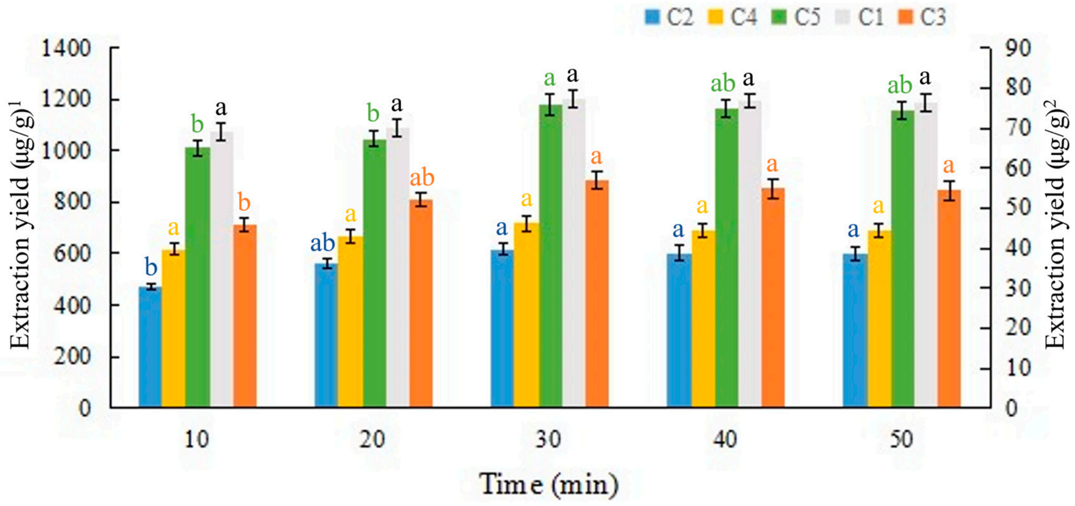

Figure S4. Effect of extraction time on the yields of five target macamides. Different lowercase in the same color of column represent the significant difference at $p < 0.05$. ([1] Extraction yields of C2, C4 and C5; [2] Extraction yields of C1 and C3).

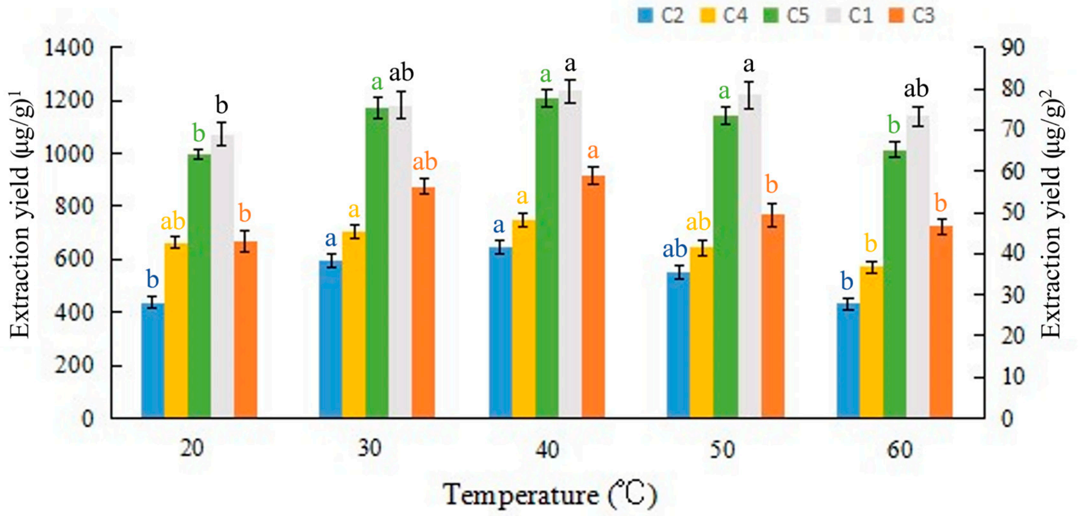

Figure S5. Effect of extraction temperature on the yields of five target macamides. Different lowercase in the same color of column represent the significant difference at $p < 0.05$. ([1] Extraction yields of C2, C4 and C5; [2] Extraction yields of C1 and C3).

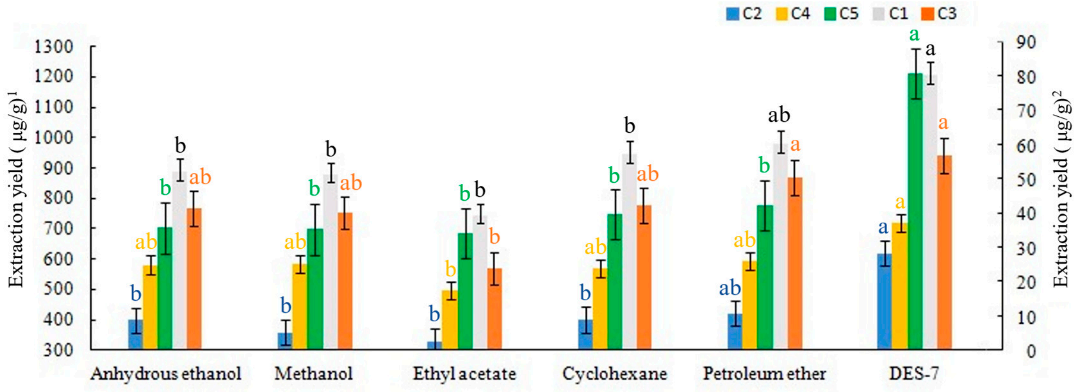

Figure S6. Comparison of the extraction efficiency between DES and conventional organic solvents by UAE. Different lowercase in the same color of column represent the significant difference at $p < 0.05$. ([1] Extraction yields of C2, C4 and C5; [2] Extraction yields of C1 and C3).

Table S1 Linear regression, LODs, LOQs, precisions, stability and repeatability of the four analytes by HPLC-DAD.

Analytes	Regression equations	R^2	Linear ranges (μg/mL)	LODs (ng)	LOQs (ng)	Intra-day precision (n = 6, RSD%)	Inter-day precision (n = 3, RSD%)	Stability (n = 3, RSD%)	Repeatability (n = 6, RSD%)
C1	Y = 14.490X - 0.4902	0.9993	1.30—82.57	2.57	7.79	2.41	1.86	1.36	1.64
C2	Y = 20.290X + 78.308	0.9994	3.52-600.00	1.46	4.38	3.83	3.98	4.48	1.92
C3	Y = 19.989X + 11.205	0.9999	0.84-200.00	2.06	6.37	1.79	2.66	3.12	1.58
C4	Y = 18.460X - 16.961	0.9992	8.00-2000.00	1.64	4.96	3.55	4.77	4.58	1.71
C5	Y = 13.045X - 23.197	0.9996	4.00-2000.00	2.82	8.56	3.68	3.79	3.22	2.05

Table S2 The optimal conditions by DESs-UAE for the five macamides.

Term	C1	C2	C3	C4	C5
X_1 (mL/g)	9.60	9.32	9.40	9.64	9.44
X_2 (°C)	38.6	38.5	38.3	38.6	37.7
X_3 (min)	28.7	25.8	27.2	26.8	27.2
Yields (μg/g)					
Predicted	79.57	617.92	57.32	714.45	1226.81
Observed	79.27	608.53	56.67	708.61	1212.89
δ (%)	0.38	1.54	1.15	0.82	1.15
